# Multifunctional platinum nanoparticles from *Chlorella vulgaris*: a statistical optimization study

**DOI:** 10.1186/s13568-026-02041-5

**Published:** 2026-04-10

**Authors:** Noura Salah Nour, Ahmed Atef El-Beih, Sawsan Abd Ellatif, El-sayed Mahdy, Hatem El-Mezayen

**Affiliations:** 1 Bioprocess Development Department, Genetic Engineering and Biotechnology Research Institute (GEBRI), City of Scientific Research and Technological Applications (SRTA-City), New Borg El-Arab, 21934 Alexandria Egypt; 2 Chemistry of natural and microbial products Department, National Research Centre, 33 El- Bohouth St., Dokki, Giza, 12622 Egypt; 3 Biochemistry division, Chemistry Department, Helwan University, Cairo, Egypt

**Keywords:** *Chlorella vulgaris*, Platinum nanoparticles, Wound healing, Nanozyme, Antioxidant, Box-Behnken design

## Abstract

This study reports the statistical optimization of platinum nanoparticles synthesized using intracellular polysaccharides from *Chlorella vulgaris* via a Box-Behnken design. Platinic acid and polysaccharide concentrations were identified as the dominant factors controlling nanoparticle formation, while temperature showed no statistically significant effect within the tested range. Model validation under optimal conditions (400 µM platinic acid, 200 µM polysaccharides, 97 ℃) confirmed strong agreement between predicted and experimental responses (R² = 0.968, *p* = 0.012), supporting model reliability. The optimized CV-PtNPs (23.1 nm) exhibited potent peroxidase and oxidase-like nanozyme activities with strong substrate affinity toward H_2_O_2_ and TMB, highlighting their catalytic efficiency. In addition, the nanoparticles demonstrated moderate antibacterial activity against Gram-positive and Gram-negative pathogens, including methicillin-resistant *Staphylococcus aureus* (MRSA), as determined by agar diffusion assays. Additional assays indicated moderate antioxidant activity and enhanced wound closure, presented here as secondary biological effects supporting their multifunctional potential. These findings demonstrate the value of statistical design in achieving reproducible control over the catalytic properties of biologically synthesized platinum nanoparticles.

**Graphical Abstract:**

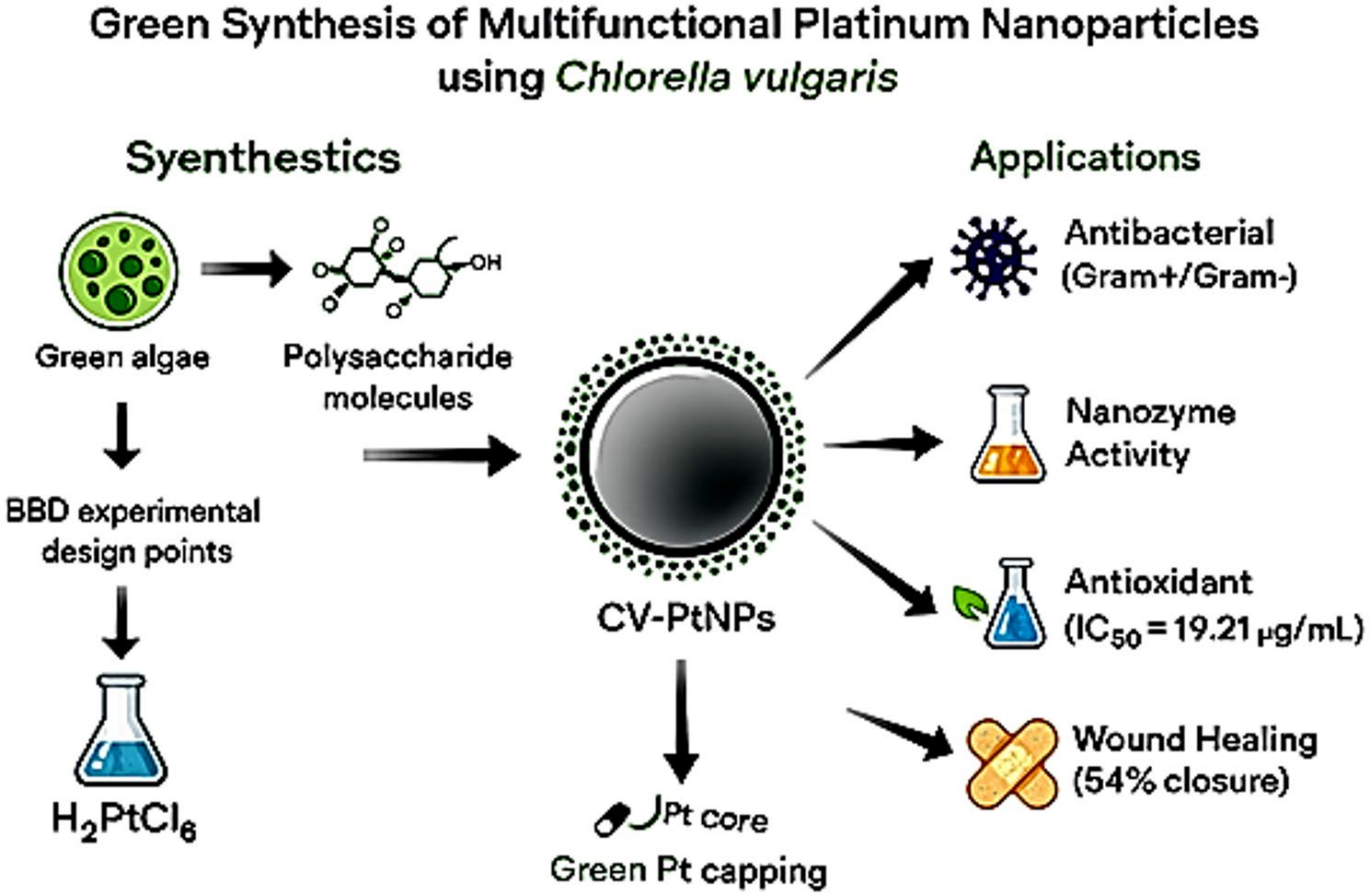

**Supplementary Information:**

The online version contains supplementary material available at 10.1186/s13568-026-02041-5.

## Introduction

Nanotechnology has enabled new biomedical strategies. It provides new approaches to drug delivery, treatments of infections, and diagnostics (Khan et al. 2019). Platinum nanoparticles have gained significant attention among various nanomaterials; they have strong efficacy in diverse applications including catalysis, sensing, and biomedicine (Pedone et al. 2017; Shurygina and Shurygin 2017; Jan et al. 2021; Gutierrez et al. 2022). Their biomedical potential primarily arises from nanozyme activity and antibacterial activities, with wound healing and antioxidant properties providing additional supportive functions (Gao et al. 2007; Shurygina and Shurygin 2017; Rajendran et al. 2020; Manzoor et al. 2021; Liu et al. 2022).

However, conventional methods of synthesizing PtNPs often rely on toxic reducing agents and high energy input, leading to environmental concerns and reduced biocompatibility (Jeyaraj et al. 2019; Cardoso et al. 2025). These challenges have driven the development of green synthesis strategies that are more sustainable, cost-effective, and biologically compatible.

Biological resources, such as plants, bacteria, and microalgae, offer eco-friendly platforms for nanoparticle synthesis (Noah and Ndangili 2022; Shah et al. 2025). Microalgae, particularly *Chlorella vulgaris*, are promising bio-factories due to their rapid growth rates and abundant bioactive compounds including proteins, pigments and polysaccharides. Intracellular polysaccharides play a dual role as both reducing and stabilizing agents, facilitating controlled nanoparticle formation while ensuring colloidal stability and functional performance (Michalak and Chojnacka 2015; Coronado-Reyes et al. 2020; Dolganyuk et al. 2020; Alqarni et al. 2022; Arenas et al. 2025).

Despite these advantages, challenges remain in optimizing key synthesis parameters such as precursor concentration, pH, temperature, and reaction time (Rehman et al. 2022a, b). Traditional one-variable-at-a-time (OVAT) approaches are inefficient and fail to capture interactions between factors that critically influence nanoparticle size, shape, and functionality (Birla et al. 2025). Response surface methodology, particularly Box-Behnken design, provides a robust statistical framework for identifying optimal conditions, minimizing experimental effort, and refining nanoparticle properties (Ferreira et al. 2007; Rehman et al. 2022a, b).

In this study, we report a green and statistically optimized synthesis of CV-PtNPs using intracellular polysaccharides of *Chlorella vulgaris*. The primary aim was to optimize synthesis parameters through a Box-Behnken design and to evaluate the resulting nanoparticles for nanozyme catalytic and antimicrobial activities. Secondary objectives included assessing antioxidant potential and in vitro wound healing efficacy to explore their broader biomedical applicability. To the best of our knowledge, this is the first study to report the integrated multifunctional behavior of statistically optimized algae-derived CV-PtNPs, combining nanozyme activity (peroxidase- and oxidase-like) with antibacterial effects, while additional bioassays are presented as supportive evaluations of functional biocompatibility.

## Materials and methods

### Chemicals and test strains

Hexachloroplatinic acid (H_2_PtCl_6_.5H_2_O) and 3, 3’, 5, 5’-tetramethylbenzidine (TMB) from (Sigma-Aldrish, St. Louis, MO, USA), the algal strain *Chlorella vulgaris* ESA251 was obtained from SRTA-City, Egypt. The strain has been deposited in the Culture Collection of Ain Shams University (CCASU, WDCM 1186), Cairo, Egypt, where it is publicly available under permanent accession number (CCASU-2025-F20). A deposit certificate confirming the availability of living cultures is provided as Supplementary Material. The corresponding GenBank sequence accession number is (PX603247), *Klebsiella pneumoniae* ATCC 700,603, *Pseudomonas aeruginosa* ATCC 27,853, *E. coli* ATCC 25,922, methicillin susceptible *staphylococcus aureus (*MSSA ATCC 25923) and methicillin resistant *staphylococcus aureus (*MRSA ATCC 43300) were obtained from SRTA-City, Egypt.

### Extraction of *C. vulgaris* polysaccharides

Intracellular polysaccharides were extracted from *C. vulgaris* ESA251 by adapted hot water extraction process. In short, freeze-dried algal biomass (10 g) was dispersed in 200 mL of distilled water and autoclaved at 121 ℃ for 30 min. The supernatant was obtained after centrifugation (10,000 × g, 20 min), concentrated by rotary evaporation and precipitated with three volumes of ice-cold ethanol. The polysaccharide precipitation was harvested, lyophilized, and stored at -20℃ (Yu et al. 2019).

### Optimization of platinum nanoparticles production by Box-Behnken design (BBD)

After biosynthesis, CV-PtNPs characterization and identification of key factors through screening and extraction of *C. vulgaris* polysaccharides, a Box-Behnken design was employed to optimize CV-PtNPs using three independent factors: platinic acid concentration, polysaccharide concentration and temperature, based on preliminary screening experiments (Francis et al. 2003). The tested ranges were 150–400 µM H_2_PtCl_6_, 50–200 µM algal polysaccharide extract, 70–100 ℃, corresponding to coded levels (-1,0,+1), and were used to fit a polynomial model (Eq. 1):

1 $$ \begin{aligned} \user2{Y}\, & = \,\user2{\beta }0 + \user2{\beta }1\user2{x}1 + \user2{\beta }2\user2{x}2 + \user2{\beta }3\user2{x}3 + \user2{\beta }12\user2{x}1\user2{x}2 \\ & + \user2{\beta }13\user2{x}1\user2{x}3 + \user2{\beta }23\user2{x}2\user2{x}3 + \user2{\beta }11\user2{x}1\user2{x}1 \\ & + \user2{\beta }22\user2{x}2\user2{x}2 + \user2{\beta }33\user2{x}3\user2{x}3 \\ \end{aligned} $$

Where Y represents absorbance intensity. β0 is the intercept factor, β1–β3 are linear coefficients, β12–β13 are interactive coefficients, and β11–β33 are quadratic coefficients, and X1, X2, X3 are coded independent variables. These independent variables were present in 15 combinations, as marked by the symbol code, actual levels of these variables and the absorbance intensity and Predicted absorbance intensity values, as shown in Supplementary Table S1. Factors to be explored and their levels in the experimental design, symbol code and actual variable level are shown in Supplementary Table S2.

### Model validation

Optimized levels of variables were experimentally validated and plotted against the theoretical values.

### Characterization of CV-PtNPs

UV-Vis Spectroscopy: Absorption spectra (200–800 nm) were recorded using a Shimadzu UV-2700 spectrophotometer. Absorbance intensity at 215 nm was used here as a relative empirical indicator under identical synthesis conditions. This band lies within the UV region where platinum nanoparticles exhibit absorption associated with interband electronic transitions (5d → 6sp) rather than a sharp visible surface plasmon resonance peak (Cele et al. 2018). In this study, the reproducible feature at 215 nm is attributed to such interband transitions, potentially influenced by contributions from *C. vulgaris* polysaccharides (Jeon et al. 2023). Under controlled, identical conditions, variations in the intensity of this band were consistently associated with relative changes in nanoparticle yield, supporting its use as the response variable for statistical optimization.

Transmission Electron Microscopy (TEM): A drop of diluted CV-PtNPs suspension was placed on a carbon-covered copper grid and allowed to air dry. Imaging was carried out using a JEOL GEM-1010 Transmission electron microscope at 80 kV (Amin et al. 2021). Particle size analysis was performed using ImageJ software (NIH, USA). The scale was calibrated using the embedded 100 nm scale bar. A total of *n* = 50 well-resolved, individual nanoparticles were manually measured from TEM images by drawing a line across the longest particle axis. Aggregated and unclear particles were excluded from analysis. Mean particle diameter, standard deviation, median size, and size range were calculated.

Energy Dispersive X-ray (EDX): Thermo-Noran EDX attachment was used for elemental analysis (Scimeca et al. 2018).

Fourier Transform Infrared Spectroscopy (FTIR): Spectra of pure polysaccharides extract and CV-PtNPs were recorded using a [shimadzu FTIR-8400s] in the 400–4000 cm range using KBr method (Pasieczna-Patkowska et al. 2025).

### Catalytic and antimicrobial performance of CV-PtNPs

#### Antimicrobial test

The antibacterial activity of CV-PtNPs was assessed based on a previously reported method (Tahir et al. 2017). Bacterial cultures were grown in nutrient broth at 37 ℃ for 24 h. The bacterial suspensions were uniformly spread onto agar plates using a sterile swab. Wells (6 mm in diameter) were created in the agar using a sterile cork borer, and 50 µL of CV-PtNPs solution (1 mg/mL) was added to each well. Ciprofloxacin was used as a positive control. The plates were incubated at 37 ℃ for 24 h, after which the zones of inhibition were measured.

### Nanozyme activity assays

#### Peroxidase-like activity of biosynthesized platinum nanoparticles

Peroxidase-like activity was evaluated by monitoring TMB oxidation at 652 nm. The CV-PtNPs concentration used was 10 µM, and the experiments were carried out in an acetate buffer (pH 4).

To investigate the peroxidase-like activity of the Pt nanoparticles; various concentrations of H_2_O_2_ were employed keeping that of TMB constant and various concentrations of TMB were employed keeping that of H_2_O_2_ constant. Then, a known volume of the CV-PtNPs solution was added to the working solution to trigger the reaction. To ensure the accuracy of the results, every experiment was performed in triplicate. Concentrations were determined from absorbance data using the Beer-Lambert Law, with a molar absorption coefficient of 39,000 M^−1^cm^− 1^ applied to TMB-derived oxidation products. Initial velocities (v) were calculated from absorbance changes and fitted to the Michaelis-Menten model to determine kinetic parameters describing the catalytic performance of CV-PtNPs Eq. 2 (Zhang et al. 2010).

2 $$\mathrm{V}\,\mathrm{=}\,\mathrm{V}\mathrm{max}\frac{\text{}\left[\mathrm{S}\right]}{\mathrm{Km}\mathrm{+}\left[\mathrm{S}\right]}$$

The values for Vmax and Km were also calculated from the Lineweaver-Burk plot using the following Eq. 3 (Li et al. 2015).

3 $$\mathrm{1/}\mathrm{V}\,\mathrm{=}\,\mathrm{Km}\mathrm{/}\mathrm{Vmax}\mathrm{[}\mathrm{S}\mathrm{]+1/}\mathrm{Vmax}$$

#### Oxidase-like activity

60 µL of 0.75 mg/mL concentration CV-PtNPs dissolved in deionized water was added to 140 µL of TMB in a 2.8 mL acetic acid-acetate buffer at pH 4 and temperature 35℃. The measurements were performed for the given time duration using a spectrophotometer to record the absorbance spectrum at 652 nm. Lineweaver-Burk plots were utilized to obtain the Michaelis-Menten constants (Cheng et al. 2020).

### In vitro antioxidant activity (DPPH scavenging assay)

The free radical scavenging activity of the CV-PtNPs biosynthesized was examined with the 2,2- diphenyl- 1- picrylhydrazyl (DPPH) assay employing a previously reported procedure with some modifications (Brand-Williams et al. 1995). Briefly, 0.1 mM ethanol solution of DPPH was prepared. The CV-PtNPs sample was serially diluted in ethanol to have concentrations ranging from 1000 µg/mL to 1.95 µg/mL. For assay, 1 mL of DPPH solution was mixed with 3 mL of each sample concentration, vortexed and incubated in the dark at room temperature. For 30 min, after which absorbance was measured at 517 nm. Ascorbic acid was used as standard. Ethanol was used as control. Scavenging activity against DPPH was calculated as % inhibition by the following formula:

4 $$DPPH~\,scavenging~effect~\% ~ = ~A0{-}A1/A0\,\times \,100$$

where A0 is the control absorbance, A1 is sample absorbance. The experiment was performed in triplicate. IC_50_ was determined from the graph drawn between scavenging % and sample concentration.

### Scratch assay test evaluation of biosynthesized CV-PtNPs for wound-healing activity

The effect of CV-PtNPs on wound healing in experiments was evaluated using the scratch assay. Surfaces of dermal cell lines were scratched by a pipette tip to form an artificial wounded area on the cell line. Later, images of cell migration in the process of wound healing were taken (Fushimi et al. 2012). In this research, these cell lines were collected from the Cairo Cancer Institute, Egypt. Under the guidance of Cormier et al. the cell culture was performed in six multi-well plates to confluences (Cormier et al. 2015). A linear scratch was performed using a sterilized and 30-degree angled yellow micro pipette tip in order to ensure uniform scratch width. The scratch was later treated with platinum nanoparticle for 48 h. The reciprocal scratch edges were then examined using a 10X objective lens. Based on the formula given below, the percentage of wound closure in comparison to that of the original scratch was calculated:

5 $$ \begin{gathered} \user2{Wound~healing} \hfill \\ \, = \left( {\user2{area~of~wound}\left( {\user2{t} = 0\user2{h}} \right) - \user2{area~of~wound}\left( {\user2{t} = 48\user2{h}} \right)} \right) \hfill \\ \;/\user2{area~of~wound}\left( {\user2{t} = 0\user2{h}} \right)\user2{*}100 \hfill \\ \end{gathered} $$

### Statistical analysis

BBD was carried out with the assistance of STATISTICA 8 (Stafsoft, Inc.) and analysis of variance (ANOVA) for model fitting and significance testing. All the experiments with biological and catalytic assays were performed in triplicate (*n* = 3) and data are presented as mean ± SD.

## Results

### Box-Behnken design and model fitting

To systematically determine the optimal conditions for synthesizing CV-PtNPs, we used a Box-Behnken Design (BBD). This method efficiently tests how three key synthesis variables, labeled x1, x2 and x3, and their interactions affect the absorbance intensity at 215 nm, which was used as the response variable for statistical optimization (see Methods).

We performed 15 specific synthesis runs, each with a different combination of the three variables. For every run, we analyzed the product by UV-Vis spectroscopy. Supplementary Fig. S1 shows the complete set of UV scans from all 15 experiments.

The order of experiments was randomized to ensure statistical reliability. The actual absorbance intensity values measured at 215 nm, along with the conditions for each run and the model’s predicted absorbance intensity values, are compiled in Supplementary Table S1.

To establish a quantitative relationship between the synthesis variables and the absorbance intensity, we performed a multiple regression analysis on this data. This produced a predictive second-order polynomial model. The final Eq. 6, expressed in uncoded actual units, is as follows:

6 $$ \begin{aligned} \user2{Response~Y}\, & = \,2.2005 + 0.929875\user2{x}1 + 0.337\user2{x}2 \\ & - 0.02113\user2{x}3 - 0.004\user2{x}1\user2{x}1 - 0.13375\user2{x}2\user2{x}2 \\ & - 0.142\user2{x}3\user2{x}3 - 0.23025\user2{x}1\user2{x}2 - 0.0725\user2{x}1\user2{x}3 \\ & + 0.31725\user2{x}3\user2{x}2 \\ \end{aligned} $$

Where x1, x2, x3 are the platinic acid concentration, polysaccharides concentration, temperature, respectively. The regression statistics and ANOVA results analysis indicated that model was statistically significant (F = 13.82, *p* = 0.012), confirming the adequacy of the quadratic model. The model showed a strong agreement between experimental and predicted values (R^2^ = 0.968; adjusted R^2^ = 0.8987), indicating reliable prediction performance. The relatively low standard error of 0.263 also supports the reliability of the regression fit.

**Fig. 1:**
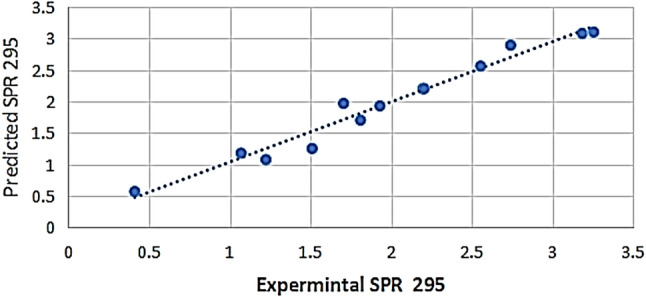
The parity plot showing agreement between Predicted and experimental responses for CV-PtNPs synthesis under Box-Behnken design conditions

Regression coefficients analysis Table 1 indicated that x1 (*p* = 0.00056) and x2 (*p* = 0.0222) linear effects were statistically significant, with the maximum positive effect produced by x1. However, x3 had no significant effect (*p* = 0.831). All the quadratic terms (x12, x22, x32) were not significant (*p* > 0.3), indicating minimal curvature in the response surface. Amongst the interaction terms, x2 × 3 exerted a nearly significant influence (*p* = 0.073), while x1 × 2 and x1 × 3 interactions were statistically insignificant (*p* > 0.15). Collectively, these results indicate that the response was mainly governed by the linear contributions of x1 and x2. Non-linear interactions contributed minimally. Adequacy of the model was further confirmed by similarity in the agreement between experimental and predicted values (Fig. 1), confirming its suitability for optimization. Analysis of the Pareto chart (Supplementary Fig. S2) was used to determine the order of factors affecting CV-PtNPs concentration. The hierarchy of influencing variables from highest to lowest impact as follows: platinic acid concentration (x1)> algal polysaccharides concentration (x2) > interaction between polysaccharides concentration and temperature interaction (x2 × 3) > interaction between the platinic acid concentration and polysaccharides concentration (x1 × 2) > quadratic effect of temperature (x3^2^) > quadratic effect of polysaccharides concentration (x2^2^) > interaction between platinic acid concentration and temperature (x1 × 3) > temperature (x3) > quadratic effect of platinic acid concentration (x1^2^).

**Table 1:** Statistical analysis of key variables using Box-Behnken design (BBD)

	Coefficient	*P*-value	Lower 95% CI	Upper 95% CI
Intercept	2.201	0.000	1.685	2.716
x1	0.930	0.001	0.672	1.188
x2	0.337	0.022	0.079	0.595
x3	-0.021	0.831	-0.279	0.237
x1^2^	-0.004	0.980	-0.412	0.404
x2^2^	-0.134	0.414	-0.541	0.274
x3^2^	-0.142	0.388	-0.550	0.266
x1 × 2	-0.230	0.154	-0.595	0.134
x1 × 3	-0.073	0.610	-0.437	0.292
x2 × 3	0.317	0.073	-0.047	0.682

### Response surface analysis

**Fig. 2:**
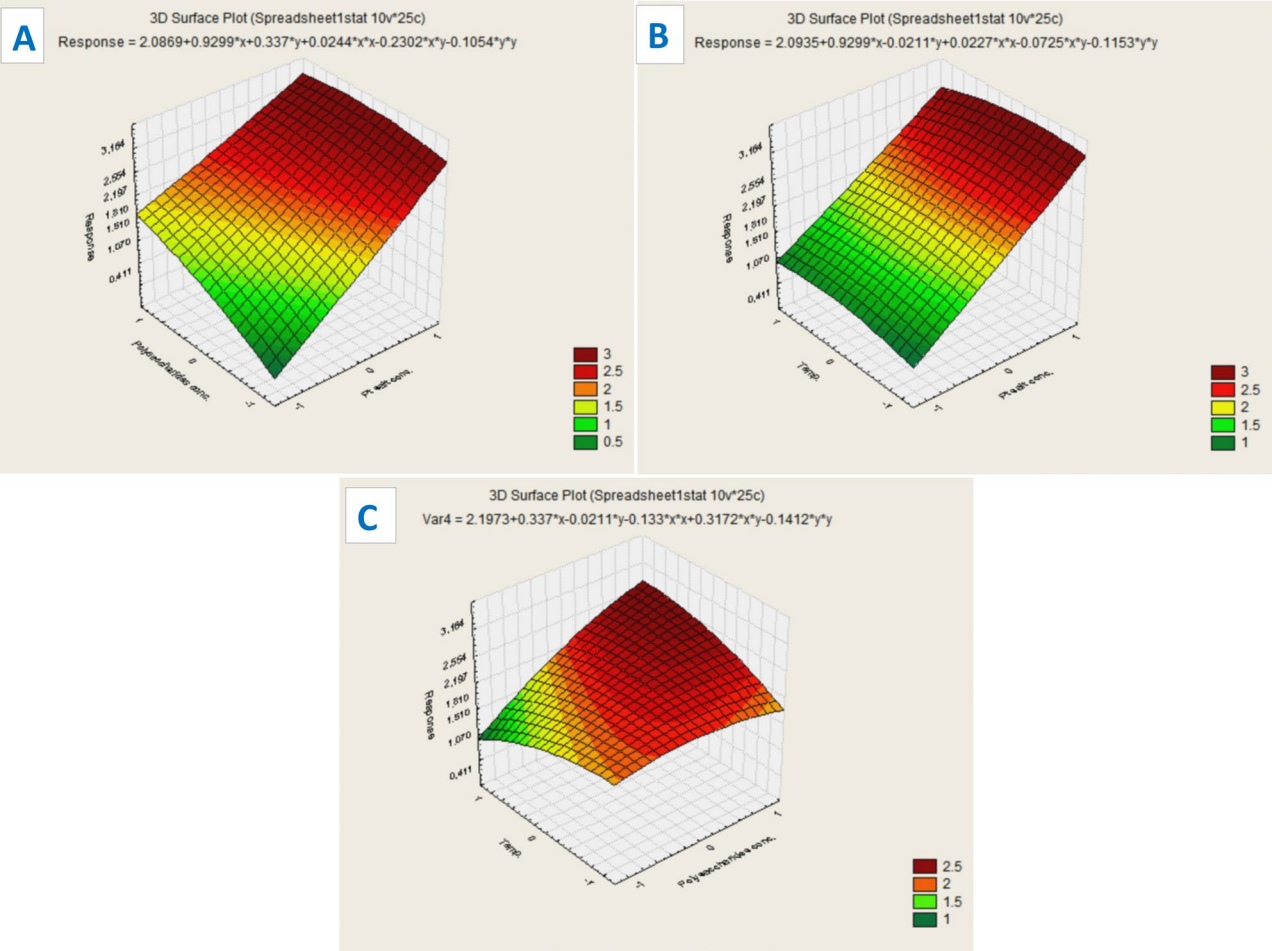
3D response surface plots showing interaction effects of synthesis variables on CV-PtNPs formation: **A** polysaccharides concentration vs. platinic acid salt concentration, **B** Temperature vs. platinic acid concentration, and **C** Temperature vs. polysaccharides concentration

To visualize the interactions among the independent variables, 3D surface and contour plots were generated using STATISTICA 8. The surface plot, (Fig. 2), of x1 (concentration of platinic acid) and x2 (concentration of polysaccharides) at constant x3(temperature) showed the strongest effect, where the increase in both variables leads to a marked increase in the response, in agreement with their high positive linear coefficients (Fig. 2A). In contrast, the interaction between platinic acid and temperature (Fig. 2B) produced a relatively flat surface, confirming the non-significant effect of temperature. Similarly, the interaction between polysaccharide concentration and temperature (Fig. 2C) showed only slight curvature, in agreement with the near-significant interaction effect observed in the regression analysis (*p* = 0.073). All three surface plots together demonstrate that the concentration of platinic acid and the concentration of polysaccharides are the primary parameters determining nanoparticles synthesis, while temperature is less important. The absence of strong curvature further verifies that the response is mainly governed by linear effects, in line with the regression analysis results.

### Experimental validation of the model

Model validation experiments performed under optimized conditions showed close agreement between predicted and experimental responses, confirming model reliability.

### Physicochemical characterization of optimized CV-PtNPs

**Fig. 3:**
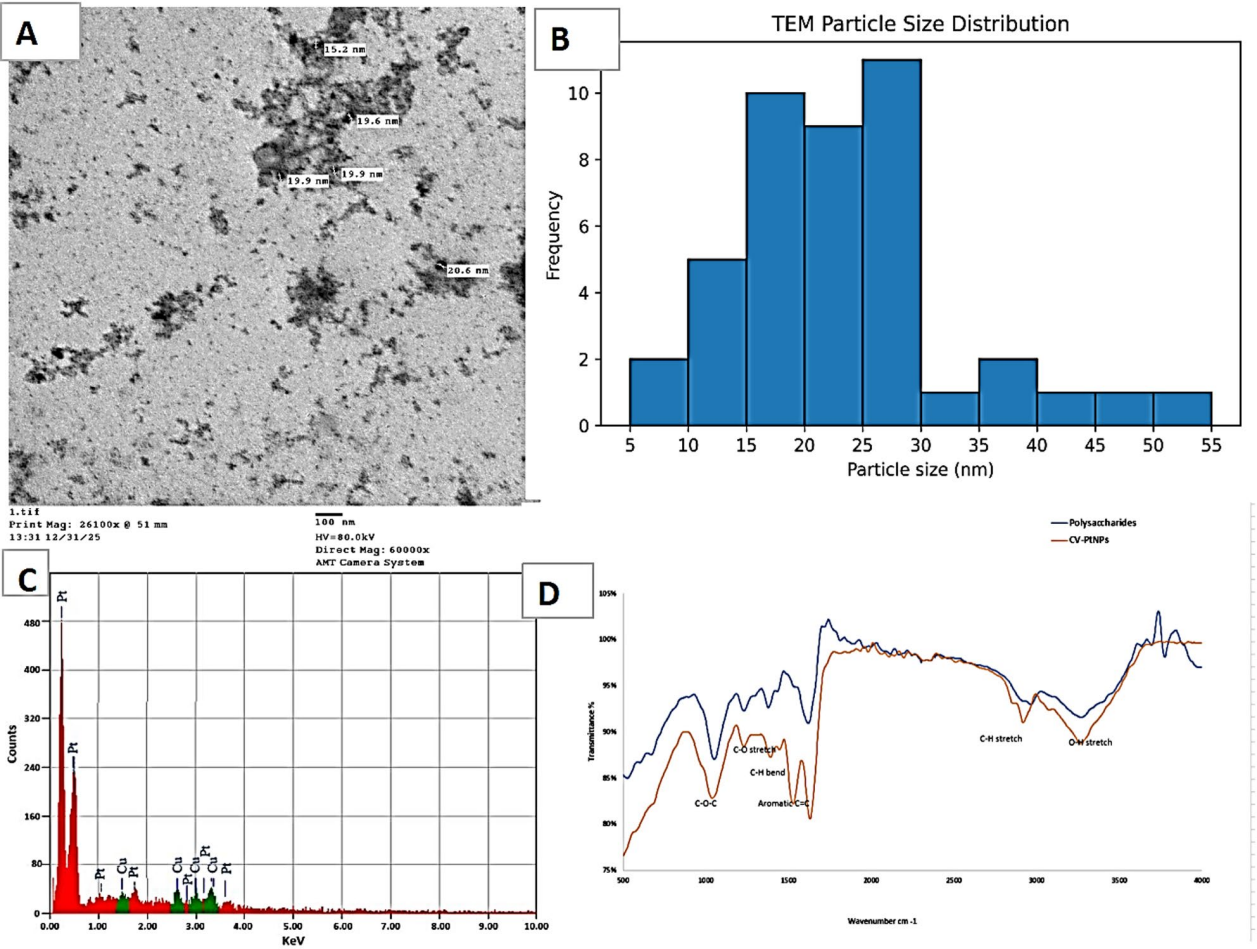
Characterization of optimized biosynthesized platinum nanoparticles (CV-PtNPs). **A** TEM micrograph showing well-dispersed, spherical CV-PtNPs (scale bar: 100 nm). **B** Particle size distribution. **C** EDX spectrum confirming platinum and associated biogenic elements. **D** FTIR spectra of CV-PtNPs (orange) and *C. vulgaris* polysaccharides (blue) (400–4000 cm⁻¹)

The morphology and size of the synthesized nanoparticles were examined by TEM (Fig. 3A). The TEM image revealed predominantly spherical nanoparticles with good dispersion, indicating limited aggregation. Quantitative analysis of 50 particles using ImageJ software showed a mean diameter of 23.1 ± 9.4 nm (range: 8.1–51.6 nm), and a median of 20.6 nm. The size distribution histogram (Fig. 3B) indicated a primary population centered around 20 nm and a smaller secondary population of larger particles (~ 40 nm). The particle size distribution showed moderate polydispersity. Morphological analysis showed a mean circularity of 0.91 ± 0.05 and aspect ratio of 1.12 ± 0.18, confirming most nanoparticles were nearly spherical with slight variations.

EDX spectrum (Fig. 3C) confirmed the presence of elemental platinum, supporting successful nanoparticle formation and indicating minimal contamination from residual biomolecules.

FTIR analysis provided evidence for the involvement of biomolecules from *Chlorella vulgaris* in both the reduction and stabilization of CV-PtNPs (Fig. 3D). The broad O-H stretching band around ~ 3400 cm^-1^ exhibited a noticeable shift and reduction in intensity after nanoparticle formation, indicating the participation of hydroxyl groups as electron donors in the reduction of Pt^+ 4^ ions (Dai et al. 2023). Similarly, the C = O stretching band at ~ 1650 cm^-1^ became weaker and slightly shifted, suggesting the involvement of carbonyl functionalities in redox processes (saito et al. 2022). The attenuation of bands near ~ 1400 cm^-1^ (COO^-^) further supports their role in electrostatic interactions with nanoparticle surface. In addition, the broadening of the C-O-C stretching region (1000–1150 cm^-1^), characteristic of glycosidic linkages, indicates that polysaccharides act as capping agents, contributing to nanoparticle stabilization (Nikoneko et al. 2000). Detailed peak assignments and comparative analysis are provided in Supplementary Table S3.

### Catalytic and antimicrobial performance of CV-PtNPs

#### In-vitro antibacterial activity

The biosynthesized CV-PtNPs were evaluated for their antibacterial activity against a panel of clinically relevant Gram-positive and Gram-negative pathogens. As illustrated in (Supplementary Fig. S3), the CV-PtNPs exhibited moderate antibacterial activity as determined by the agar well diffusion assay, producing measurable zones of inhibition.

The zones of inhibition ranged from approximately 11 to 13 mm across all tested strains. The most susceptible bacterium was the Gram-negative *Klebsiella pneumoniae*, exhibiting a zone of 13 mm, whereas the Gram-positive methicillin-susceptible *Staphylococcus aureus* (MSSA) showed the lowest susceptibility with a zone of 11 mm. A standard broad-spectrum antibiotic (ciprofloxacin) was employed as a positive control. As anticipated, the standard antibiotic exhibited significantly larger inhibition zones, ranging from 23 to 25 mm for all strains.

#### Peroxidase-like activity of biosynthesized CV-PtNPs

**Fig. 4:**
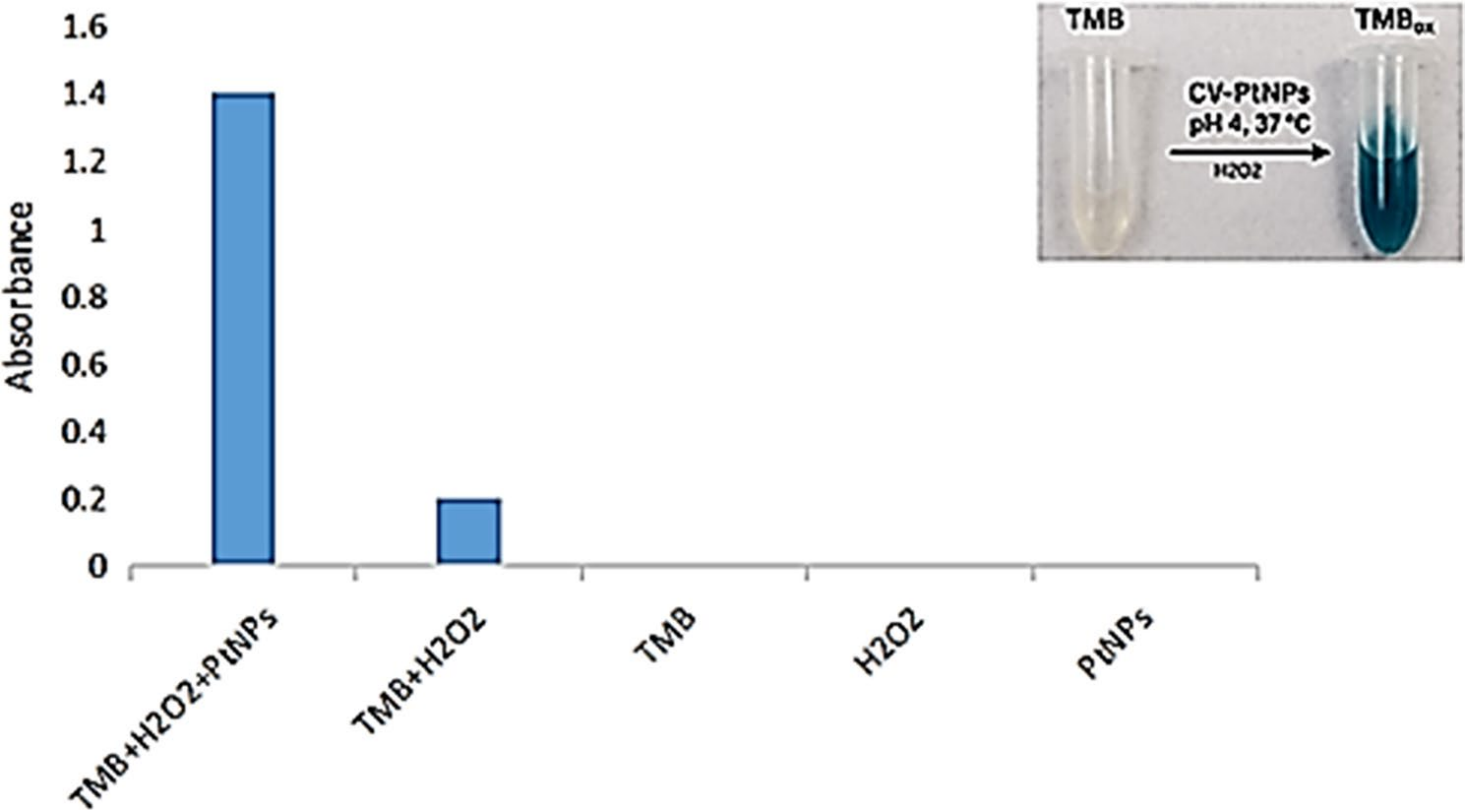
Peroxidase-like activity of CV-PtNPs monitored by TMB oxidation in the presence of H₂O₂ at 652 nm. Inset: color change associated with oxidized TMB formation

CV-PtNPs exhibited peroxidase-like activity, evidenced by rapid oxidation of TMB in the presence of H_2_O_2_, producing a characteristic blue color (Fig. 4).

Catalytic efficiency was evaluated by fitting initial reaction rates to the Michaelis-Menten model, allowing calculation of Km and Vmax values.

At constant saturating TMB, varying H₂O₂ concentration gave Km = 0.349 mM and Vmax = 13.67 µM s⁻¹ (R² = 0.969). Upon altering the concentration of TMB at a constant, saturating concentration of H₂O₂, the kinetics provided a Km of 3.601 mM and a Vmax of 16.40 µM s⁻¹ (R² = 0.988), as indicated in (Supplementary Fig. S4). These values indicate catalytic performance comparable to that of natural horseradish peroxidase (HRP) and other representative nanozymes (Supplementary Table S4).

### Oxidase-like catalytic activity and kinetic analysis

Oxidase-like activity was confirmed by catalytic oxidation of TMB, following Michaelis-Menten kinetics (R^2^= 0.9941), indicating efficient substrate binding.

**Fig. 5:**
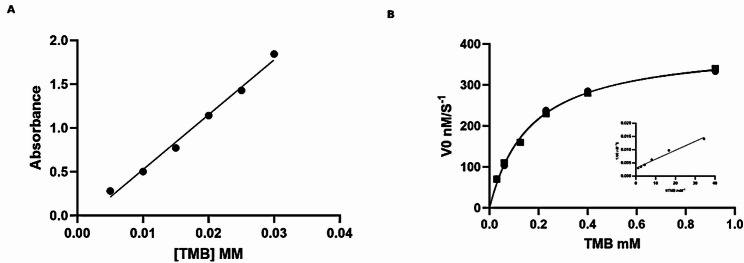
Oxidase- like activity of CV-PtNPs. **A** Variation of absorbance with TMB concentration. **B** Michaelis–Menten with corresponding Lineweaver–Burk inset

To evaluate the CV-PtNPs’ catalytic behavior, initial reaction rates (V₀) were determined at different TMB concentrations and fitted to the Michaelis-Menten model (Fig. 5). The model showed a good fit, with a Km of 0.16 mM and a Vmax of 0.395 µM s⁻¹ (Table 2). The low Km value indicates high substrate affinity. Linear Lineweaver-Burk analysis supported the calculated kinetic parameters (Fig. 5B).

Compared to reported oxidase-like nanozymes, CV-PtNPs demonstrated competitive catalytic efficiency (Table 3). Overall, these kinetic analyses confirm that CV-PtNPs function as oxidase-like nanozyme.

**Table 2:** Kinetic parameters of CV-PtNPs as oxidase- like nanozyme for TMB oxidation

Parameter	Value	95% confidence interval
Vmax (µM s⁻¹)	0.395	0.36–0.436
Km (mM)	0.16	0.12–0.21
R²	0.9941	

**Table 3:** Comparison of kinetic parameters of CV-PtNPs with reported nanozymes

Nanozyme	Vmax (nM s⁻¹)	Km (mM)	Reference
CV-PtNPs	**395.1**	**0.16**	This work
Fe₃O₄ NPs	~ 180	0.71	(Wang et al. 2018)
Citrate-AuNPs	~ 95	0.43	(Shah and Singh 2018)
PVP-AgNPs	~ 150	0.38	(Abdel-Lateef et al. 2022)
CeO₂ NPs	~ 220	0.55	(Asati et al. 2009)

### In vitro antioxidant activity

**Fig. 6:**
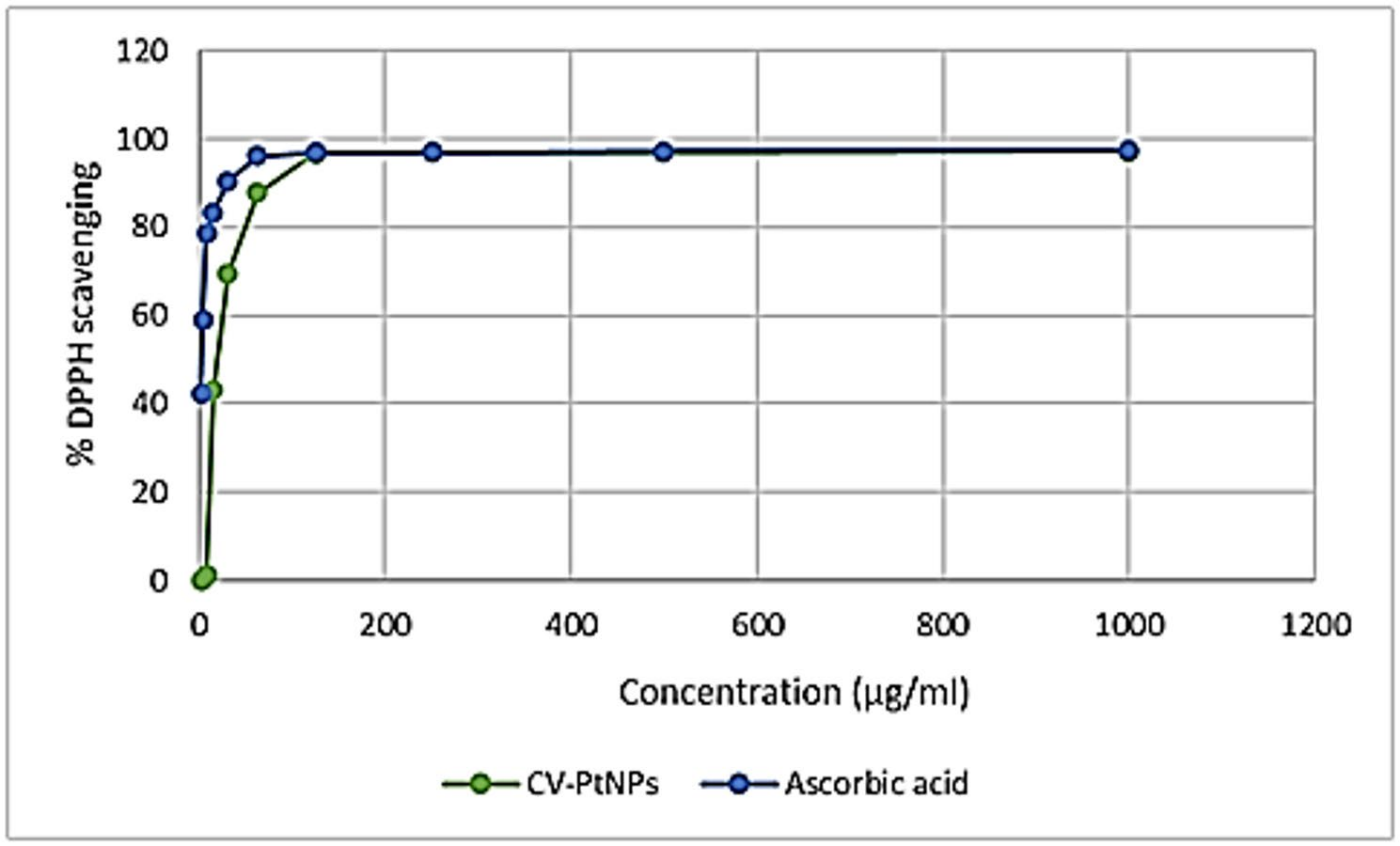
DPPH scavenging efficacy of CV-PtNPs and Ascorbic acid at different concentrations

The antioxidant activity of CV-PtNPs was evaluated using DPPH assay (Supplementary Table S5, Fig. 6). CV-PtNPs showed concentration-dependent radical scavenging activity with an IC_50_ of 19.21 µg/mL, indicating moderate antioxidant potential as a secondary biological effect.

### Wound healing activity of CV-PtNPs

**Fig. 7:**
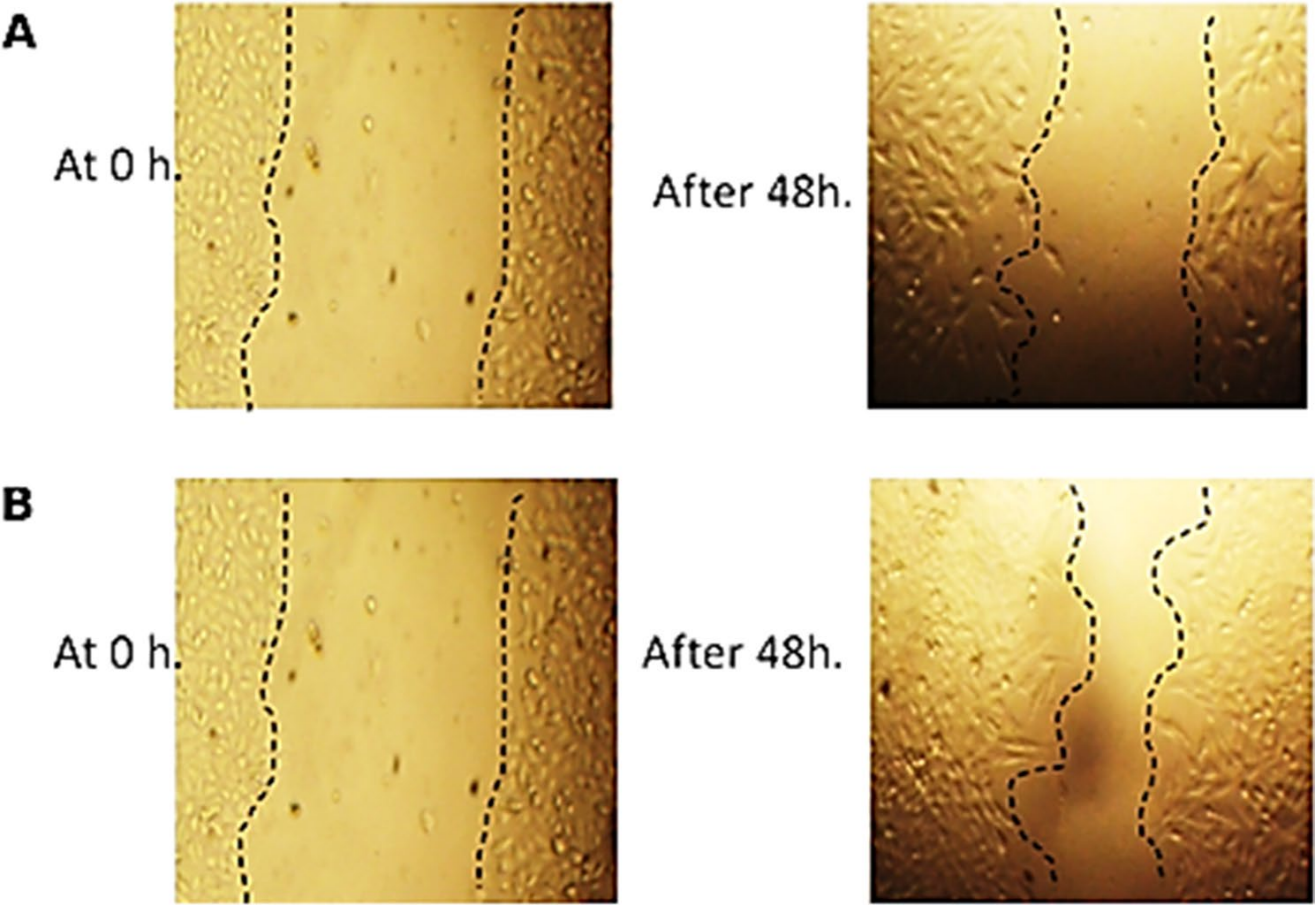
Wound healing activity of (**A**) control and (**B**) CV-PtNPs at different time intervals

As a complementary biological evaluation, a scratch assay was performed to assess wound closure (Fig. 7). Quantitative analysis showed a statistically significant increase in closure in the CV-PtNPs treated group (54.39 ± 7.18%) compared to the control (30.04 ± 4.56%) after 48 h (*p* < 0.001). A reduction in wound width and an increase in area reduction were also observed (Supplementary Table S6). Data are presented as mean ± SD (*n* = 6), and statistical significance was determined using an unpaired Student’s t-test.

## Discussion

Our study established a green and statistically guided strategy for synthesizing platinum nanoparticles (CV-PtNPs) using *C. vulgaris* polysaccharides as both reducing and stabilizing agents. Box-Behnken design identified precursor concentration as the primary determinant of nanoparticle formation (Szabó et al. 2022). Most quadratic terms were statistically non-significant, consistent with the predominantly linear response observed, indicating that the model’s strength lies in identifying dominant synthesis variables rather than curvature-driven effects.

Temperature exerted only a minor effect within the tested range. This contrasts with conventional chemical synthesis, where temperature strongly influences nucleation dynamics (Lee et al. 2023). This observation supports the hypothesis that biomolecular capping agents may play a dominant role in directing nanoparticle formation under biogenic conditions (Javed et al. 2020). Reduced temperature sensitivity may simplify process scalability by reducing the need for strict thermal control. The optimized synthesis conditions likely contributed to improved catalytic performance through controlling nanoparticle size and dispersion, which are known determinants of nanozyme activity (Meng et al. 2023).

Our comprehensive physicochemical characterization confirmed successful nanoparticle formation. The TEM results demonstrated the formation of ~ 23 nm spherical nanoparticles, consistent with biologically synthesized CV-PtNPs reported (Ullah et al. 2017). The FTIR evidence of polysaccharide binding explains the efficient colloidal stability observed during storage and biological testing.

The optimized CV-PtNPs exhibited antibacterial activity. The inhibition zones obtained using agar diffusion assays reflect apparent activity but may be influenced by limited nanoparticle diffusion in solid media (Pelgrift and Friedman 2013). Although minimum inhibitory concentration (MIC) and minimum bactericidal concentration (MBC) were not determined in this study, the observed inhibition zones provide preliminary evidence of antibacterial activity, warranting further quantitative evaluation. Reported mechanisms, including ROS generation (Godoy-Gallardo et al. 2021) and membrane disruption(Dzuvor et al. 2024), may contribute to the observed effects, although these pathways were not directly tested in this study. Such multimodal mechanisms may, in principle, reduce the likelihood of rapid resistance development (Wang et al. 2021a, b; Zhou et al. 2023), although this requires further validation.

An important finding was the nanozyme behavior of optimized CV-PtNPs. The nanoparticles showed high affinity for H₂O₂ (with a Km of 0.349 mM), which is higher than that of natural horseradish peroxidase (Veitch 2004) and better than many other synthetic nanozymes, including CeO₂ and carbon-based ones. Their oxidase-like activity was similarly competitive. This enhanced catalytic behavior may be associated with polysaccharides-mediated stabilization, which could facilitate electron transfer during catalytic reactions (Allawadhi et al. 2022), although this mechanism was not directly confirmed. Collectively, these results demonstrate the role of statistical optimization in enhancing catalytic functionality.

The observed antioxidant activity may contribute to biological compatibility. However, this function is considered secondary to the nanozyme activity emphasized in this study. Further mechanistic investigations are needed to elucidate the relationship between catalytic properties and the associated biological responses.

## Conclusion

In summary, this work demonstrates that *C. vulgaris* polysaccharides are an efficient, eco-friendly tool for synthesizing multifunctional platinum nanoparticles on a scalable basis. The optimized synthesis produced CV-PtNPs exhibiting nanozyme activity and measurable antibacterial effects. Although antioxidant and wound healing responses were observed, the primary strength of CV-PtNPs lies in their nanozyme and antimicrobial performance.

Collectively, these findings position statistically optimized algae-derived CV-PtNPs as a promising platform for nanozyme-assisted antimicrobial applications. Future studies should elucidate the molecular mechanisms underlying nanoparticle-biological interactions, particularly the role of the polysaccharide coating and evaluate therapeutic efficacy and long-term safety in in vivo. Integration into advanced delivery systems, such as smart wound dressings, represents a potential translational direction.

## Supplementary Information

Supplementary Material 1.

## Data Availability

All data generated or analyzed in this study are presented within the manuscript.
