# Supplementary material for: Multifunctional platinum nanoparticles from Chlorella vulgaris: a statistical optimization study
Source: AMB Express. 2026 Apr 10;16:45. doi: 10.1186/s13568-026-02041-5 (PMC13083553; doi:10.1186/s13568-026-02041-5)
Supplement: Supplementary file 1 — Supplementary Material 1. [file 13568_2026_2041_MOESM1_ESM.docx]

**Supplementary Table S 1** Box-Behnken design matrix with coded factor levels, observed SPR intensity, and model-predicted response for CV-PtNPs optimization

| Run | x1 | x2 | x3 | spr intensity | Predicted spr intensity |
| --- | --- | --- | --- | --- | --- |
| 1 | 0 | 0 | 0 | 2.197 | 2.2005 |
| 2 | -1 | -1 | 0 | 0.411 | 0.565625 |
| 3 | 0 | 1 | 1 | 2.554 | 2.557875 |
| 4 | 0 | -1 | 1 | 1.51 | 1.249375 |
| 6 | 1 | 1 | 0 | 3.254 | 3.099375 |
| 7 | 1 | 0 | 1 | 2.74 | 2.89075 |
| 8 | 1 | 0 | -1 | 3.184 | 3.078 |
| 9 | -1 | 1 | 0 | 1.81 | 1.700125 |
| 10 | -1 | 0 | 1 | 1.07 | 1.176 |
| 11 | 0 | 1 | -1 | 1.705 | 1.965625 |
| 12 | 0 | -1 | -1 | 1.93 | 1.926125 |
| 13 | -1 | 0 | -1 | 1.224 | 1.07325 |
| 14 | 0 | 0 | 0 | 2.204 | 2.2005 |
| 15 | 1 | -1 | 0 | 2.776 | 2.885875 |

**Supplementary Table S 2** The true values of the coded variables of Box-Behnken design

| **Level** | **H_2_PtCl_6_**  **Volume (μL)** | **Algal polysaccharides volume (μL)** | **Temp (^o^C)** |
| --- | --- | --- | --- |
|  | **x1** | **x2** | **x3** |
| 1 | 400 | 200 | 100 |
| 0 | 200 | 100 | 90 |
| -1 | 150 | 50 | 70 |

**Supplementary Table S 3** FTIR peak assignments of C. vulgaris intracellular polysaccharides and CV-PtNPs with proposed functional roles in reduction and stabilization

| **Wavenuber (cm^-1^)** | **Assignment** | **Polysaccharides** | **CV-PtNPs** | **Interpretation** |
| --- | --- | --- | --- | --- |
| ~3400 | O-H stretching (hydroxyl groups) | Strong, broad | Shifted and reduced | Hydroxyl groups donate electrons, reduce Pt4+ |
| ~1650 | C=O stretching (carbonyl groups) | Strong | Shifted and weaker | Carbonyl groups involved in reduction of Pt ions |
| ~1400 | C-H bending/ COO- vibrations | Moderate | Reduced | COO- participates in electrostatic interaction |
| 1000-1150 | C-O-C stretching (glycosidic linkages) | strong | Broadened | Ether linkages stabilize PtNPs by capping |
| ~850 | Polysaccharide skeletal vibrations | Present | reduced | Indicates binding to nanoparticle surface |

**Supplementary Table S 4** Kinetic parameters of CV-PtNPs for H₂O₂ and TMB compared with natural horseradish peroxidase (HRP) and other representative nanozymes

| **Catalyst** | **Substrate** | **K_m_ (mM)** | **V_max (μM s⁻¹)** | **Reference** |
| --- | --- | --- | --- | --- |
| **CV-PtNPs** | H₂O₂ | 0.349 | 13.67 | This work |
| (this work) | TMB | 3.601 | 16.40 | This work |
| **HRP**(natural) | H₂O₂ | ~3.7 | — | (Veitch 2004) |
|  | TMB | ~0.434 | — | (Veitch 2004) |
| **Fe₃O₄ NPs** | H₂O₂ | ~1.0–1.5 | <10 | (Wu et al. 2014) |
| **AuNPs** | H₂O₂ | ~1.2 | <12 | (Song et al. 2010) |

**Supplementary Table S 5** Antioxidant activity of CV-PtNPs compared with ascorbic acid standard as measured by the DPPH free- radical scavenging assay.

| **Concn. (μg/mL)** | **Ascorbic acid** | | | **CV-PtNPs** | | |
| --- | --- | --- | --- | --- | --- | --- |
|  | **Absorbance** | **Scavenging %** | **IC_50_** | **Absorbance** | **Scavenging %** | **IC_50_** |
| **1000** | 0.015 | 97.37 | 2.92 | 0.017 | 97.14 | 19.21 |
|  |  |  |  |  |  |  |
| **500** | 0.017 | 97.14 |  | 0.017 | 97.08 |  |
| **250** | 0.017 | 97.08 |  | 0.018 | 96.91 |  |
| **125** | 0.018 | 96.96 |  | 0.018 | 96.85 |  |
| **62.5** | 0.022 | 96.16 |  | 0.071 | 87.74 |  |
| **31.25** | 0.056 | 90.44 |  | 0.177 | 69.59 |  |
| **15.62** | 0.096 | 83.45 |  | 0.330 | 43.30 |  |
| **7.81** | 0.124 | 78.69 |  | 0.573 | 1.49 |  |
| **3.9** | 0.240 | 58.82 |  | 0.580 | 0.29 |  |
| **1.95** | 0.335 | 42.50 |  | 0.581 | 0.11 |  |

**Supplementary Table S 6** Wound healing assay of CV-PtNPs at different time intervals

| **Parameter** | **CV-PtNPs (Mean ±SD)** | **Control(Mean ±SD)** | **p-value** |
| --- | --- | --- | --- |
| **Width at 48 (µm)** | 484 ± 83.46 | 743.07 ± 52.58 | <0.001 |
| **Wound closure (%)** | 54.39 ± 7.18 | 30.04 ± 4.56 | <0.001 |
| **Area difference (µm^2^)** | 621379 ± 70984 | 343187±41629 | <0.001 |


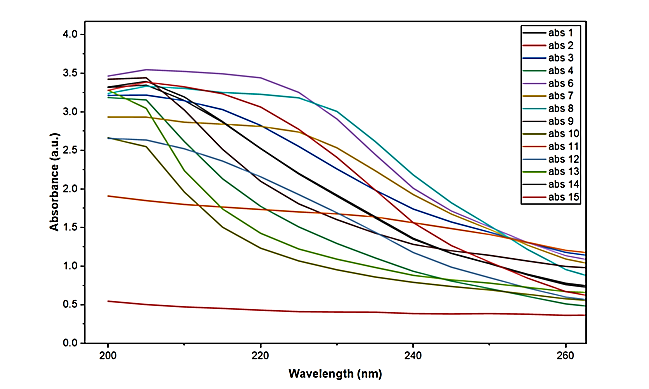


**Supplementary Fig. S 1** UV scan for 15 runs of Box-Behnken design for biosynthesis of Pt Nanoparticles using C. Vulgaris


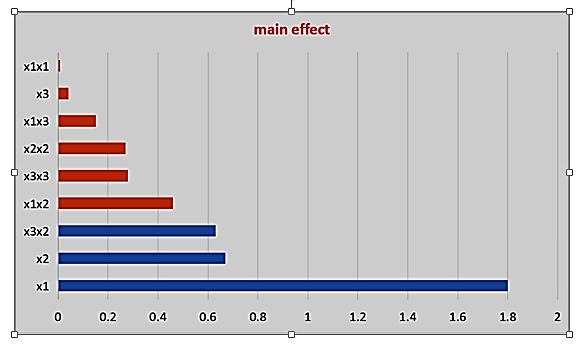


**Supplementary Fig. S 2** Graph of Pareto analysis ranking effectiveness of individual variables on CV-PtNPs SPR (blue columns affect positively while orange columns affect negatively).


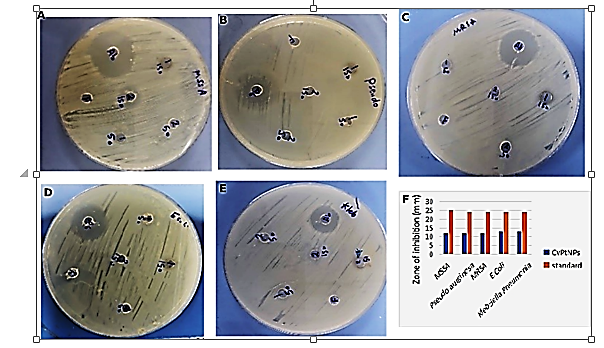


**Supplementary Fig. S 3** Antibacterial performance of CV-PtNPs by agar well diffusion method against different pathogenic strains (A) MSSA, B) *Pseudomonas aeruginosa*, C) MRSA, D) *Escherichia coli*, and E) *Klebsiella pneumoniae*). F) Comparison with standard antibiotic. The results are expressed as the mean zone of inhibition (mm)


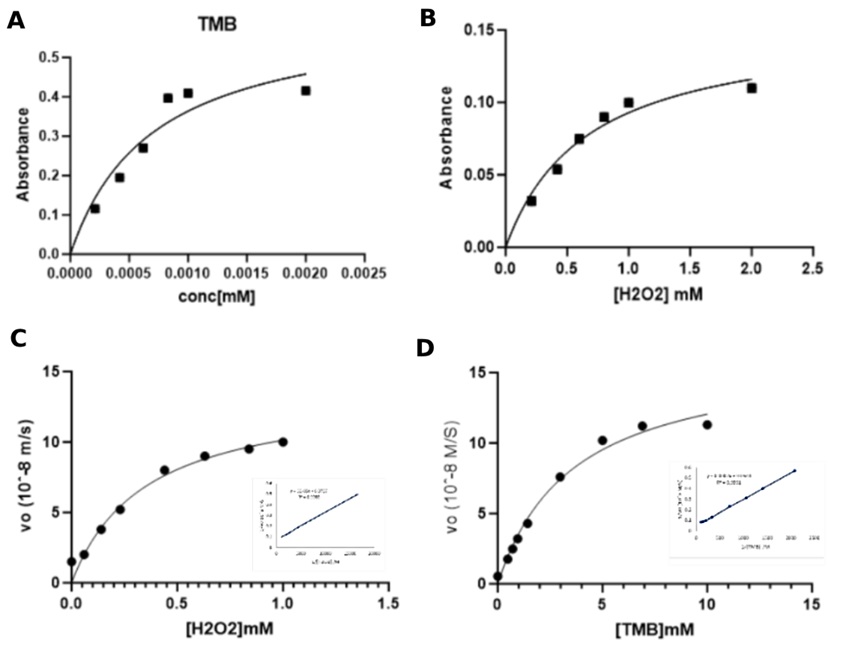


**Supplementary Fig. S 4** Steady state kinetics assay of CV-PtNPs with peroxidase- like activity. (A)Variation of absorbance with TMB concentration. (B) Variation of absorbance with H_2_O_2_ concentration, demonstrating substrate-dependent catalytic activity. (C,D) Michaelis–Menten plots showing the catalytic activity of C. vulgaris-mediated platinum nanoparticles (CV-PtNPs) toward (C) hydrogen peroxide (H_2_O_2_) and (D) 3,3′,5,5′-tetramethylbenzidine (TMB). Insets represent the corresponding Lineweaver–Burk plots
